# A Systematic Review of Interventions That Reduce Family Caregiving Time

**DOI:** 10.1093/geroni/igab046.1034

**Published:** 2021-12-17

**Authors:** Zachary Baker, Eric Jutkowitz, Joseph Gaugler

**Affiliations:** 1 University of Minnesota, University of Minnesota/Minneapolis, Minnesota, United States; 2 Brown University, Brown University, Rhode Island, United States; 3 University of Minnesota, Minneapolis, Minnesota, United States

## Abstract

Due to multiple long-term sociodemographic and health trends contributing to the impending family care gap, there likely is no single policy or intervention that could increase the number of family caregivers in the U.S. to the levels required to fill such a gap. However, the amount of time that a family caregiver spends providing assistance is potentially mutable. Given the pressing concerns of the family care gap, identifying interventions or approaches that could reduce existing caregiving time is of considerable importance. This presentation provides the results of a systematic review of published research to identify the effects of interventions on the amount of time family caregivers spend on their caregiving tasks. Pharmaceutical approaches directed to care recipients, technology interventions, case management, multicomponent interventions, and care settings all appeared to reduce caregiving time. Improved operationalization, study design, and similar factors will help guide future intervention research to reduce caregiving time.

